# The Association Between Power of Food Scale Scores and Weight Among Black/African American Individuals Consuming a Vegan or Low‐Fat Omnivorous Diet in a Randomized Controlled Trial

**DOI:** 10.1155/jnme/4084525

**Published:** 2026-04-09

**Authors:** Alexis Bell, John A. Bernhart, Yesil Kim, Enid A. Keseko, Gabrielle Turner-McGrievy

**Affiliations:** ^1^ Department of Health Promotion, Education, and Behavior, University of South Carolina, Columbia, South Carolina, 29208, USA, sc.edu; ^2^ Prevention Research Center, University of South Carolina, Columbia, South Carolina, 29208, USA, sc.edu

**Keywords:** appetite, Black or African American, cardiovascular diseases, hunger, weight loss

## Abstract

The NEW Soul study was a 2‐year, 2‐group randomized controlled trial intervention in South Carolina comparing the effects of a vegan (plant based) diet to those of a low‐fat (< 30%) omnivorous diet among African American adults, ages 18–65 years old, and with overweight or obesity. The current study assessed changes in the Power of Food Scale (PFS) scores—a measure of hedonic hunger using three domains (food available, food tasted, and food present)—by group across the study and the relationship between changes in PFS scores and weight loss among all participants such that increases in PFS scores were associated with less weight loss. We used repeated measures models to compare changes in scores by group. We also used repeated measures models to see if changes in scores were associated with changes in weight, controlling for employment status, education, food security status, sex, and age. There were no differences in PFS scores between the vegan and low‐fat omnivorous groups across time; however, both groups showed decreases in PFS domain scores over time suggesting reduced hedonic hunger among all participants. Furthermore, while score changes in the food available and food tasted domains were not associated with weight change, significant associations were observed for the food present domain and the total PFS scores (*p* < 0.05 for both). Future studies should more closely target food available, tasted, and present domains to see if changes are associated with weight loss.

**Trial Registration:** ClinicalTrials.gov identifier: NCT03354377

## 1. Introduction

Changes to lifestyle habits and committing to these changes long term to encourage weight loss can be challenging. Furthermore, improving dietary intake can have a significant impact on the promotion of weight loss, but improving dietary intake and maintaining those changes can be difficult [1–3]. Barriers to adopting healthier diets for general populations can include distance to grocery stores, lack of transportation, selection of items available at grocery stores, and quality of items at grocery stores, as well as insufficient social support and health status, such as chronic disease risk factors, which have been shown to influence diet quality and perceived barriers to healthier eating [4–6]. Specifically for African American (AA) populations, barriers to healthier eating that have negative impacts on their diet include cost of food and available finances, availability and convenience, absence of fruits and vegetables in the home, and lack of knowledge regarding healthy foods [7–10].

An additional major barrier with dietary change is hedonic hunger [11, 12]. Hedonic hunger is characterized as the motivation to consume food for pleasure versus consuming food to eliminate hunger [11]. While hedonic hunger may be experienced by most individuals to some extent, emotional eating—the tendency to overeat in response to negative emotions—may further influence consumption and contribute to the development and maintenance of obesity. For some individuals, hedonic hunger is an issue that can lead to unnecessary overconsumption of foods [13]. Motivations that may encourage hedonic hunger include advertisements, smelling food, and seeing others eat [13]. Also, for some individuals, the physiological impact of food influences what they eat. For example, research has shown that the release of dopamine occurs when individuals ingest palatable foods [14]. When this release occurs, it can encourage individuals to consume more foods due to the excessive incentive salience they experience, causing them to overeat [14].

In order to measure appetite for food among different populations, the Power of Food Scale (PFS) was developed [15]. The PFS is a measure of individual differences in appetite‐related thoughts, feelings, and motivations in environments where plentiful palatable foods are constantly available [15]. Previous research on the PFS measure has shown that hedonic hunger is related to motivation to consume highly palatable foods in the absence of physiological hunger due to increased response in visual food cues and anticipation of reward received from consumption of palatable foods [13]. Previous research has also found that the PFS measures appetitive drive to consume palatable foods more strongly than measuring the tendency to consume increased amounts of food [13]. Also, related to the PFS measure, research suggests that PFS scores decrease over time for individuals who participate in behavioral weight loss or surgical treatment for overweight/obesity due to volitional changes in eating behaviors and major changes in appetitive hormones, respectively [13].

The use of the PFS in behavioral interventions and randomized clinical trials is limited, but previous interventions have used the measure to study cravings for specific foods or impacts of food cravings on weight loss [16–18]. For example, in one study examining two different weight loss strategies, a control‐based coping strategy and an acceptance‐based strategy, researchers found that higher scores on the PFS were predictive of greater cravings and food consumption for both groups [16]. Previous research has also found that participants in different behavioral weight loss treatment conditions who report high levels of hedonic hunger experience less weight loss than those with lower hedonic hunger scores [19]. While the impact that hedonic hunger has on populations has been studied among predominantly white populations in the United States (U.S.), research on the relationship between hedonic hunger with weight loss among AA adults participating in behavioral nutrition interventions is limited.

The purpose of this paper is to examine potential behavioral benefits related to hedonic hunger and motivation to consume palatable foods, as assessed by the PFS, between participants randomized to either the vegan diet that emphasized minimally processed whole foods from plants or low‐fat omnivorous soul food diets—a traditional diet originating in the southern U.S. and ethnic to AA adults—and to assess the relationship between changes in PFS scores and weight loss across the 24‐month study. Objectives related to harms were not examined. Because both groups participated in a similar behavioral weight loss intervention that included strategies for adopting healthy foods, we hypothesized that there would be no differences between groups in changes in PFS scores across time and decreases in PFS scores would be associated with decreases in weight.

## 2. Methods

### 2.1. Study Design and Participants

The randomized clinical trial Nutritious Eating with Soul (NEW Soul) study was a 24‐month nutrition intervention aimed to examine the impact of soul food‐focused vegan and omnivorous diets on changes in risk factors for cardiovascular disease (CVD) among AA adults residing in the southeast U.S. and was registered [20]. The trial protocol and statistical analysis plans of this study have been described elsewhere [20]. Participants were eligible for the study if they self‐identified as AA; were between 18 and 65 years old; had a BMI between 25 and 49.9 kg/m^2^; live in the Columbia, SC area; were able to attend all monitoring visits; willing to be randomized to either diet; free of major health or psychiatric diseases, drug or alcohol dependency; free of an eating disorder as screened by the Eating disorder Screen for Primary care (ESP); and free on either of the two meeting nights (e.g., Monday or Wednesday). Regarding recruitment, participants were recruited through various methods, including media interviews, radio commercials, community outreach, and word of mouth [21]. Before the intervention, participants were informed up front about what the study involved. To keep participants motivated throughout the study, participants were incentivized for class attendance (through weekly class drawings) and for completion of assessments (through gift card incentives). The two randomized dietary intervention groups: a vegan or omnivorous diet, focused on the consumption of soul food cuisine [20]. The primary parameter of the present study and secondary analysis was to examine changes in PFS scores overtime, including both domain specific and total scores. Sample size was calculated elsewhere [20].

### 2.2. Intervention Description

After randomization, participants attended nutrition classes that occurred weekly for the first 6 months of the study, biweekly for the second 6 months, and monthly for the last 12 months [18]. Intervention classes for both groups focused on nutrition topics, cooking demonstrations, discussion of successes and challenges, stress management activities, and engaging in physical activity [18]. For the nondietary recommendations, the information remained the same for both groups. A full description of the intervention content has been published elsewhere [18]. The study was approved by a university Institutional Review Board on 4/12/2017, reference number Pro0006485. All participants provided written consent before inclusion into the study, and the privacy rights of human subjects have been observed.

### 2.3. Measures

#### 2.3.1. PFS

The PFS assesses variations in thoughts, emotions, and drivers related to appetite within environments characterized by a constant abundance of tempting foods [15]. Participants completed the PFS questionnaire at baseline and 3, 6, 12, and 24 months [20]. The PFS includes three domains and 15 total items, and responses are based on a five‐point Likert scale where a 1 means “don’t agree at all” and a 5 means “strongly agree” [15]. The three domains are food available, food present, and food tasted [15]. Food available is defined as “food in the environment that is readily available, but not physically present,” referring to food that is mentally accessible, such as anticipated to be obtainable, rather than physically visible [17]. An example questionnaire item is “I think I enjoy eating, a lot more than most other people” [17]. Food present is defined as “food that is physically present, but not tasted,” referring to food that is visible but not consumed [17]. For example, “When I know a delicious food is available, I can’t help myself from thinking about having some” [17]. Food tasted is defined as “food that is first tasted, but not yet consumed,” referring to the first initial experience of tasting the food, rather than the actual consumption of the food [17]. For example, “When I eat delicious food, I focus a lot on how good it tastes” [17]. PFS scores can range from 15 to 75. For this study, scores were calculated as averages for the analyses. The psychometric properties of the scale have been presented elsewhere and have demonstrated adequate test–retest validity and internal consistency [15]. The PFS has also been validated in additional populations including women with binge eating disorder (in the Midwest U.S.) [22]; adults with overweight and obesity (from the U.S. and Canada) [23]; preadolescents and adolescents (from the Midwestern U.S.) [24]; and a diverse college sample (from undergraduate psychology courses at the University of New Mexico) [25].

#### 2.3.2. Body Weight

Weight was assessed in kilograms at all time points using a calibrated digital scale [18]. Participants were asked to wear light street clothing or change into scrubs and remove their shoes before stepping onto the scale [18]. Their weight was recorded twice, and if the two weight measurements were not within a 0.1 kg measurement, an additional measure was taken [20].

#### 2.3.3. Statistical Analysis

Data analysis was performed from November 2023 to March 2024 by a statistician who was masked to group assignments in the study. The primary aim of the study was addressed using mixed models for repeated measures, which account for missing data, with PROC MIXED in the SAS system Version 9.4. The model included PFS domain, time, and PFS domain∗time interaction adjusted for employment, education, food security status, sex, and age as covariates. Sex is typically defined as a male or female reproducing organism, based on the functions of their reproductive system. For the purpose of this study, sex was self‐reported by the participants. Least square mean differences in PFS change scores were contrasted both within and between groups. Next, in order to address the second aim of the study, analysis of covariance (ANCOVA) was performed using PROC GLM in the SAS system (Version 9.4). The model was specified to determine if changes in the scores of the three domains of the PFS over 24 months were associated with change in body weight at 24 months among all participants, controlling for the baseline weight and the same covariates used in the mixed models. As an exploratory analysis, baseline and domain PFS scores were examined as predictors of weight outcomes at 24 months. These analyses were conducted using regression models adjusted for baseline weight and relevant covariates.

## 3. Results

**FIGURE 1:**
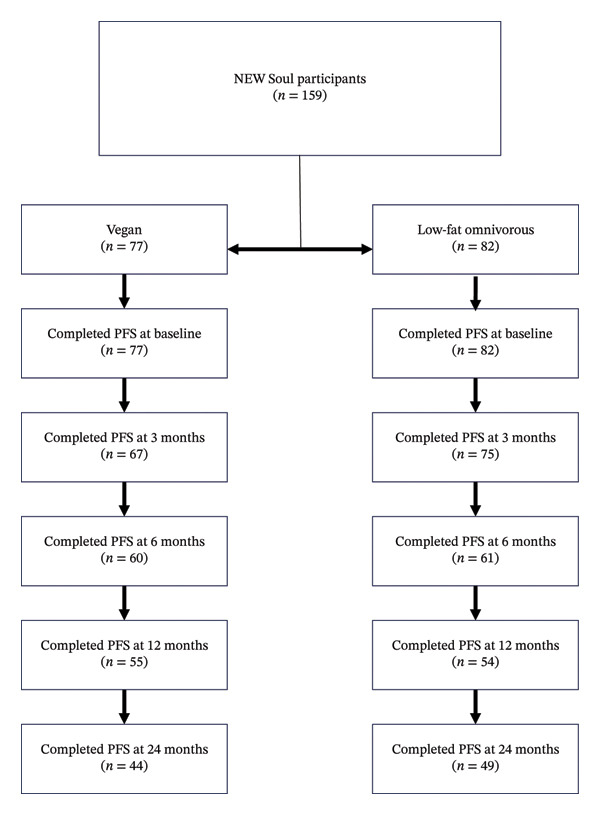
Flow diagram of participant completion of PFS questionnaire by intervention group across all study time points.

Baseline demographics have been previously published, and data were analyzed for 159 participants, which included individuals in the vegan (*n* = 77) and omnivorous (*n* = 82) groups [18]. Participants of the NEW Soul Study included mostly females (79%), had a college degree or greater (74%), were employed (74%), middle aged (mean of 48.4 years), and food secure (86%) [18]. Figure 1 shows the number of participants that completed the PFS questionnaire at each time point.

With the current study’s sample, the PFS demonstrated good internal consistency reliability for the food available domain at baseline (*α* = 0.91), 3 months (*α* = 0.90), 6 months (*α* = 0.87), and 12 months (0.89). Acceptable internal consistency reliability was demonstrated at 24 months (*α* = 0.72).

For the food present domain, good internal consistency reliability was demonstrated at baseline (*α* = 0.85), 3 months (*α* = 0.85), 6 months (*α* = 0.87), and 12 months (0.81). Acceptable internal consistency reliability was demonstrated at 24 months (*α* = 0.75).

For the food tasted domain, acceptable internal consistency reliability was demonstrated at baseline (*α* = 0.79), 3 months (*α* = 0.75), 12 months (*α* = 0.71), and 24 months (*α* = 0.76).

For the total PFS score, good internal consistency was demonstrated at all time points (*α* = 0.94 at baseline, *α* = 0.93 at 3 months, *α* = 0.91 at 6 months, *α* = 0.92 at 12 months, and 0.89 at 24 months).

Overall, weight was collected for 92% of the participants at 3 months, 89% at 6 months, 76% at 12 months, and 63% at 24 months [18].

Compliance was measured by dietary adherence (examining recommended animal product intake for 5 food groups via averaged dietary recalls at assessment time points). Adherence by group has been previously reported [18].

**FIGURE 2:**
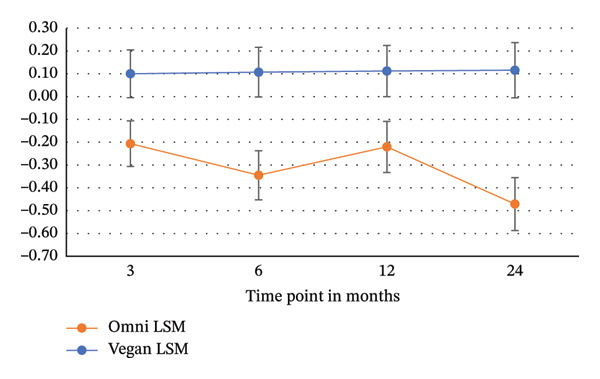
Changes in the adjusted mean for total PFS scores at all time points for the low‐fat omnivorous group and the vegan group.

Figure 2 shows changes in adjusted mean (least squares mean (LSM)) for total PFS scores at all time points (3 months, 6 months, 12 months, and 24 months) by intervention group. The between‐group differences were assessed for all time points simultaneously. Furthermore, Table 1 shows a summary of how the PFS scores changed from baseline to 3 months, 6 months, 12 months, and 24 months and presents the group by time interactions for total PFS and each of the three PFS domains. There were no statistically significant differences between groups in any of the domains. Examining within‐group changes, there were statistically significant decreases in the domain scores of food available at 6 months and 24 months for the omnivorous group and statistically significant decreases in domain scores at 3 months, 6 months, and 12 months for the vegan group. There were also statistically significant decreases in domain scores of food present at all time points for both groups. Next, there were decreases in domain scores of the food tasted domain at all time points for both groups. Lastly, for the overall PFS scores, there were also statistically significant decreases in scores for both groups at all time points. The decreases in overall scores indicate a pattern of reducing the influence of appetite‐related thoughts, feelings, and motivations on eating for all participants.

**TABLE 1:** Changes in power of food scores by participants randomized to the omnivorous or vegan diets in NEW Soul^a^ presented as adjusted means ± standard error (confidence intervals)^b^ (*n* = 159).

Change in each Power of Food Scale domain from baseline	Omnivorous (*n* = 82)	Vegan (*n* = 77)	Difference between omni and vegan
Food available			
Baseline	1.6 ± 0.2 (1.2, 2.1)	1.7 ± 0.2 (1.3, 2.1)	
3 months	−0.2 ± 0.1 (−0.4, 0.02)	−0.3 ± 0.1 (−0.5, −0.1)^∗^	−0.1 ± 0.1 (−0.4, 0.1)
6 months	−0.4 ± 0.1 (−0.6, −0.2)^∗^	−0.3 ± 0.1 (−0.5, −0.1)^∗^	0.04 ± 0.2 (−0.3, 0.3)
12 months	−0.2 ± 0.1 (−0.4, 0.03)	−0.3 ± 0.1 (−0.5, −0.1)^∗^	−0.1 ± 0.2 (−0.4, 0.2)
24 months	0.4 ± 0.1 (0.2, 0.6)^∗^	0.4 ± 0.1 (0.1, 0.6)	−0.04 ± 0.2 (−0.4, 0.3)
Food present			
Baseline	2.4 ± 0.2 (1.9, 2.8)	2.5 ± 0.2 (2.0, 2.9)	
3 months	−0.2 ± 0.1 (−0.5, −0.03)^∗^	−0.5 ± 0.1 (−0.7, −0.3)^∗^	−0.3 ± 0.2 (−0.6, 0.03)
6 months	−0.4 ± 0.1 (−0.6, −0.1)^∗^	‐‐0.5 ± 0.1 (−0.7, −0.2)^∗^	−0.1 ± 0.2 (−0.4, 0.2)
12 months	−0.3 ± 0.1 (−0.5, −0.02)^∗^	−0.5 ± 0.1 (−0.7, −0.2)^∗^	−0.2 ± 0.2 (−0.5, 0.1)
24 months	−0.7 ± 0.1 (−0.9, −0.4)^∗^	−0.7 ± 0.1 (−1.0, −0.4)^∗^	−0.03 ± 0.2 (−0.4, 0.3)
Food tasted			
Baseline	2.3 ± 0.2 (1.9, 2.7)	2.3 ± 0.2 (1.9, 2.6)	
3 months	−0.2 ± 0.1 (−0.4, −0.05)^∗^	−0.2 ± 0.1 (−0.4, −0.02)^∗^	0.02 ± 0.1 (−0.2, 0.3)
6 months	−0.4 ± 0.1 (−0.6, −0.2)^∗^	−0.2 ± 0.1 (−0.4, −0.02)^∗^	0.2 ± 0.1 (−0.1, 0.4)
12 months	−0.3 ± 0.1 (−0.5, −0.1)^∗^	−0.2 ± 0.1 (−0.4, −0.04)^∗^	0.02 ± 0.1 (−0.3, 0.3)
24 months	−0.3 ± 0.1 (−0.5, −0.1)^∗^	−0.3 ± 0.1 (−0.5, −0.1)^∗^	−0.03 ± 0.2 (−0.3, 0.3)
Total scores			
Baseline	2.0 ± 0.2 (1.6, 2.5)	2.1 ± 0.2 (1.7, 2.5)	
3 months	−0.2 ± 0.1 (−0.4, −0.01)^∗^	−0.3 ± 0.1 (−0.5, −0.1)^∗^	−0.1 ± 0.1 (−0.4, 0.1)
6 months	−0.3 ± 0.1 (−0.6, −0.1)^∗^	−0.3 ± 0.1 (−0.6, −0.1)^∗^	0.0 ± 0.2 (−0.3, 0.3)
12 months	−0.2 ± 0.1 (−0.4, 0.0)^∗^	−0.3 ± 0.1 (−0.5, −0.1)^∗^	−0.1 ± 0.2 (−0.4, 0.2)
24 months	−0.5 ± 0.1 (−0.7, −0.2)^∗^	−0.6 ± 0.1 (−0.9, −0.4)^∗^	−0.2 ± 0.2 (−0.5, 0.2)

^a^Nutritious Eating with Soul.

^b^All models were adjusted for baseline employment, education, food security status, sex, and age.

^∗^Within‐group changes *p* < 0.05.

Overall, the participants in both groups lost weight throughout the study, but there were no statistically significant differences in weight loss between the groups [18]. Table 2 shows a summary of PFS domain scores from baseline to 24 months and how changes in PFS scores impacted body weight, adjusting for employment, education, food security status, sex, and age. The score changes in the food available and food tasted domains were not associated with changes in weight. However, the score changes for the food present domain and overall total PFS scores were associated with changes in weight (*p* < 0.05 for both the food present domain and the total PFS scores).

**TABLE 2:** Association between changes in Power of Food Scale (PFS) domain scores and weight loss at 24 months among NEW Soul^a^ participants (*n* = 159).

	***b***	**SE**	**95% CI L**	**95% CI U**	***p***

*Food available by time (24 months)*					
Intercept	−0.3	8.5	−17.2	16.6	0.97
Food available × time (24 months)	0.97	0.8	−0.5	2.5	0.2

*Food present by time (24 months)*					
Intercept	−2.8	8.5	−19.7	14.1	0.74
Food present × time (24 months)	1.4	0.7	0.01	2.8	0.048^∗^

*Food tasted by time (24 months)*					
Intercept	0.3	8.7	−17.0	17.5	0.98
Food tasted × time (24 months)	0.4	0.8	−1.3	2.0	0.64

*Total scores*					
Intercept	−1.9	8.4	−18.5	14.8	0.82
Total scores × time (24 months)	0.9	0.04	0.9	1.0	< 0.0001^∗^

*Note:* All models were adjusted for employment, education, food security status, sex, and age.

^a^Nutritious Eating with Soul.

^∗^ *p* < 0.05.

Furthermore, exploratory analyses assessing baseline total and domain PFS scores as predictors of weight outcomes at 24 months indicated there were no significant associations (Table 3).

**TABLE 3:** Exploratory analysis examining baseline PFS scores as predictors of 24‐month weight outcomes among NEW Soul^a^ participants (*n* = 159).

	***b***	**SE**	**95% CI L**	**95% CI U**	***p***

*Food available by time (24 months)*					
Intercept	−3.8	8.6	−20.7	13.2	0.66
Food available × time (baseline)	−0.9	0.7	−2.3	0.4	0.18

*Food present by time (24 months)*					
Intercept	−3.3	8.5	−20.2	13.5	0.7
Food present × time (baseline)	−1.1	0.6	−2.3	0.1	0.08

*Food tasted by time (24 months)*					
Intercept	−1.9	8.9	−19.6	15.8	0.83
Food tasted × time (baseline)	−1.0	0.7	−2.4	0.5	0.20

*Total scores by time (24 months)*					
Intercept	−2.4	8.6	−19.6	14.7	0.78
Total scores × time (baseline)	−1.2	0.8	−2.7	0.3	0.11

*Note:* All models were adjusted for employment, education, food security status, sex, and age.

^a^Nutritious Eating with Soul.

## 4. Discussion

The current study hypothesized that both groups would see equal decreases in PFS scores across time and decreases in PFS scores would be associated with decreases in weight. As hypothesized, there were no differences in change in PFS scores between groups across all time points combined. One possible explanation for this finding may be that both groups’ efforts to lose weight and improve diet quality contributed to a shift in how they viewed food. It is possible that as participants focused on healthy eating behaviors, they focused more on hunger and less on hedonic cues, allowing them to make food choices based on hunger instead of pleasure. Previous research in a secondary analysis of a randomized controlled trial shows that behavioral weight loss interventions can lead to a reduction in hedonic hunger and an increase in eating behaviors that are considered healthier eating patterns (reductions in hedonic hunger, uncontrolled eating, food cravings, and obesogenic food environment) [26]. Previous research in a cross‐sectional study also shows mindful eating strategies emphasizing that physical hunger and satiety cues are associated with more healthy food choices and an overall healthy dietary pattern [27]. A second explanation for this finding is the uniformity of the information provided to both of the groups during the study. Although the groups followed two different dietary patterns, they both received the same behavioral intervention and treatment related to class topics and duration [20]. Furthermore, diet recommendations for both groups emphasized low‐fat, plant‐based foods, which also may have led to the lack of difference in weight loss seen between groups.

The present study also found that changes in PFS scores were not associated with weight loss at 24 months for the food available and food tasted domains. However, a significant association was found for the food present domain and total scores, explaining that participants with increases in food present domain scores and total scores had less weight loss/greater weight gain at 24 months. These findings partially align with the study hypothesis, as it supports the idea that hedonic hunger can interfere with an individual’s weight management. One explanation for this finding is that reducing food consumption may lead to increased food reward sensitivity as suggested by findings from an observational cohort study of U.S. emerging adults [17]. When an individual has a heightened food reward sensitivity, they may be susceptible to consuming more foods for reward, which can hinder their ability to lose weight or maintain weight loss.

Both groups reported decreases in PFS scores during the NEW Soul study at various time points, which highlights the potential benefit of participating in behavioral lifestyle nutrition interventions for AA adults on decreases in hedonic hunger. Throughout the intervention, participants received nutrition classes that focused on goal setting, discussion of successes, and challenges with dietary adoption, covering various nutrition topics, cooking demonstrations, and a physical activity or stress management activity [18]. These components could provide reasoning for why PFS scores decreased for the participants, as a previous behavioral weight loss trial study has found that individuals assigned to Weight Watchers interventions that included weekly sessions with goal‐setting, self‐monitoring, problem solving, and advice on diet, physical activity, and behavioral strategies had decreased hedonic hunger, as measured by the PFS, across a 24‐month study timeframe [12]. A second study, which was a randomized controlled trial, also found that hedonic hunger decreased and this decrease was associated with weight loss among a group of adults 25–65 years of age, who attended weekly 45‐min group meetings that included reviewing of weekly progress, discussions on weight loss, nutrition, and exercise and written materials [28].

While exploratory analyses did not identify baseline PFS scores as predictors of weight outcomes at 24 months, the observed associations with changes in the food present domain and total PFS scores over time may be more relevant for weight outcomes than baseline PFS scores. Furthermore, the results of this study can be compared with an observational cohort study that examined the PFS and its association with weight outcomes and dieting among young adults in the U.S. and a randomized controlled trial that examined the association between weight control behaviors and hedonic hunger during a 12‐week commercial weight loss program [17, 28]. Unlike a prior cohort study among young adults that found no association between PFS scores and excess weight gain [17], the present study found significant associations between food present scores and total scores, as the scores increased and there were less favorable weight outcomes at 24 months. Another randomized controlled trial of adults ages 25–65 years seeking weight loss treatment has shown that decreases in hedonic hunger, as assessed by the PFS, were associated with higher weight loss percentages [28]. The findings of the present study add to existing literature by showing that increases in scores for the food present domain and overall PFS scores were significantly associated with less weight loss/greater weight gain at 24 months. These findings suggest that hedonic hunger may interfere with an individual’s weight management. However, the NEW Soul intervention did appear to be effective in reducing hedonic hunger, as measured by decreases in PFS scores, among the participants, which highlights the potential benefit it can have for populations at risk of hedonic hunger.

The present study does have some limitations. One of these limitations includes only being able to generalize the current findings to AA residing in the southeast U.S., particularly women with high education who are employed, middle aged, and food secure. Additional limitations also include the absence of a control group that did not receive any intervention and the use of the self‐reported PFS measure. While these limitations of the study do exist, the present study does have several strengths. These strengths include utilizing objective measures to obtain weight, using a validated survey that assesses hedonic hunger among different populations, and examining changes in PFS scores over 24 months, which allowed for a long‐term examination of how PFS scores can change over time and be related to weight loss.

The findings of the current research suggest that multicomponent behavioral lifestyle nutrition interventions that place an emphasis on lifestyle behaviors and healthy eating, instead of healthy eating alone, can help inform the implementation of nutrition interventions that are targeted at reducing hedonic hunger and body weight among AA adults. These reductions can assist in alleviating obesity, especially among AA adults. Additional health benefits of reducing hedonic hunger and body weight among AA adults also include targeting the reduction of other CVD risk factors, specifically in the AA population, that can allow them to be healthier. For example, the vegan group and low‐fat omnivorous group in the NEW Soul randomized clinical trial both had improvements in weight loss and CVD risk factors among AA adults [18]. A pilot study, including a culturally tailored, community‐based mobile health lifestyle intervention was found to have an impact on improving diet and physical activity behaviors that are linked to cardiovascular health [29].

## 5. Conclusions

In this study examining differences in PFS scores and how the scores impact weight loss among a group of AA adults participating in two different behavioral lifestyle nutrition interventions, there were no differences in PFS domain changes between the vegan and low‐fat omnivorous groups. Additionally, changes in the food available and food tasted domain scores were not related to weight loss. However, increases in changes in the food present domain and total PFS scores were significantly associated with less weight loss/greater weight gain over 24 months, suggesting that how an individual responds to cues of food that is immediately visible, may be relevant for interventions targeting weight loss and the long‐term weight outcomes of individuals. Furthermore, both groups reduced PFS scores, demonstrating the intervention was associated with assisting with reducing hedonic hunger among participants. Future research should examine an intervention that more closely targets PFS domains to see if changes in these scores impact weight loss.

**Nomenclature**
AA: African Americans
PFS: Power of Food Scale
NEW Soul: Nutritious Eating With Soul
CVD: Cardiovascular disease

## Author Contributions

A.B.: conceptualization, writing–original draft, and writing–reviewing and editing; J.A.B.: data curation and writing–reviewing and editing; Y.K.: data curation, writing–original draft, and writing–reviewing and editing; E.A.K.: conceptualization, writing–original draft, and writing–reviewing and editing; G.T‐M.: conceptualization, writing–original draft, and writing–reviewing and editing.

## Funding

This work was supported by the National Heart, Blood, and Lung Institute under award project number R01HL135220.

## Disclosure

All authors are in agreement with the manuscript, and we acknowledge that this content has not been published elsewhere.

## Conflicts of Interest

The authors declare no conflicts of interest.

## Data Availability

The data and materials supporting the findings of this study are available on request from the corresponding author.
